# Cardiometabolic Index: a novel prognostic biomarker for recurrent stroke risk in acute ischemic stroke patients

**DOI:** 10.3389/fneur.2026.1803512

**Published:** 2026-06-10

**Authors:** Yue Wang, Yingying Ding, Min Han, Yehong Liu, Tao Xin

**Affiliations:** 1 Department of Neurosurgery, The First Affiliated Hospital of Shandong First Medical University and Shandong Provincial Qianfoshan Hospital, Jinan, Shandong Province, China; 2 Medical Science and Technology Innovation Center, Shandong First Medical University and Shandong Academy of Medical Sciences, Jinan, Shandong Province, China; 3 Department of Neurosurgery, The 904th Hospital of PLA, Wuxi Clinical College of Anhui Medical University, Wuxi, Jiangsu Province, China; 4 Department of Neurosurgery, The Affiliated Yixing Hospital of Jiangsu University, Wuxi, Jiangsu Province, China; 5 Department of Cardiology, The 904th Hospital of PLA, Wuxi Clinical College of Anhui Medical University, Wuxi, Jiangsu Province, China; 6 Institute of Brain Science and Brain- Inspired Research, Shandong First Medical University and Shandong Academy of Medical Sciences, Jinan, Shandong Province, China

**Keywords:** AIS, biomarker, Cardiometabolic Index, ischemic stroke, major adverse cerebrovascular events

## Abstract

**Background:**

Stroke remains a major global health problem, and recurrent stroke represents a serious complication in patients with acute ischemic stroke (AIS), which severely compromises patient prognosis and increases the socioeconomic burden on healthcare systems worldwide. Effective biomarkers for predicting major adverse cerebrovascular events (MACEs) after thrombolysis are urgently needed to optimize clinical management and improve long-term outcomes in AIS patients.

**Methods:**

This study retrospectively enrolled 588 AIS patients who underwent thrombolytic therapy. The predictive efficiency of the Cardiometabolic Index (CMI) for long-term adverse cerebrovascular events was compared with that of traditional biomarkers. Multivariate analysis was performed to identify the independent correlation between CMI and MACE risk. Additionally, the mediating effect of systemic inflammation on the association between CMI and adverse outcomes was investigated.

**Results:**

CMI demonstrated superior predictive performance for long-term adverse cerebrovascular events compared with conventional biomarkers in post-thrombolytic AIS patients. A high CMI value (≥0.80) was independently correlated with an increased risk of MACEs. Moreover, systemic inflammation partially mediated the predictive effect of CMI on adverse clinical outcomes.

**Conclusion:**

CMI is a reliable and promising prognostic biomarker for predicting adverse cerebrovascular events in AIS patients after thrombolytic therapy. By comprehensively reflecting metabolic and inflammatory status, CMI provides better predictive capability and helps refine risk stratification and individualized treatment strategies. Further large-sample cohort studies are necessary to verify the clinical applicability of CMI and improve therapeutic regimens for AIS.

## Introduction

Stroke is a leading cause of morbidity and mortality and a major cause of long-term disability (1). However, despite appropriate treatment, some patients with acute ischemic stroke (AIS) experience recurrent adverse cerebrovascular events, especially the recurrence of stroke has a great disability rate, which reduces the quality of life of patients and increases the global economic burden (2). Nonetheless, long-term follow-ups of AIS patients who have undergone emergency thrombolytic therapy indicate a prevalent occurrence of major adverse cerebrovascular events (MACEs) post-discharge, including stroke recurrence as an endpoint. These complications not only degrade the quality of life for patients but also place significant financial strains on healthcare systems worldwide (1, 2). Consequently, optimizing after intravenous thrombolysis management to accurately identify clinical predictors of MACEs and develop effective biomarkers for risk stratification and tailored treatment strategies remains a pivotal challenge.

The underlying pathogenesis of AIS primarily involves the rupture of vulnerable atherosclerotic plaques followed by thrombus formation, leading to complete occlusion of the cerebral artery and extensive cerebral infarction (3). Dysregulated lipid metabolism and immune-inflammatory responses are key contributors to the initiation and progression of cerebral artery atherosclerosis. Research indicates that stringent management of lipid levels and systemic inflammation markedly enhances clinical outcomes (4). Recent studies have underscored the prognostic significance of lipid metabolism-related biomarkers such as the triglyceride-glucose index (TyG), atherogenic index of plasma (AIP), and triglyceride-glucose body mass index (TyG-BMI) in guiding post-discharge care and improving long-term outcomes in AIS patients (5–7). Concurrently, systemic immune-inflammatory markers have gained recognition as substantial predictors of MACEs in patients of AIS after intravenous thrombolysis (8). Previous research team has shown that markers like the Systemic Inflammatory Response Index (SIRI), Pan-Immune-Inflammation Value (PIV), Neutrophil-to-Lymphocyte Ratio (NLR), Platelet-to-Lymphocyte Ratio (PLR), C-reactive Protein-to-Albumin Ratio (CAR), Neutrophil-to-Albumin Ratio (NAR), Platelet-to-Albumin Ratio (PAR), and Lymphocyte-to-C-reactive Protein Ratio (LCR) correlate with long-term outcomes (9–11). Notably, pre-procedural LCR has demonstrated superior diagnostic efficacy over other inflammatory markers (12). These findings prompt an essential inquiry: could a single biomarker that encapsulates both dysregulated lipid metabolism and systemic immune-inflammatory status offer incremental prognostic value over traditional lipid or inflammatory markers in forecasting long-term outcomes for AIS patients? This question forms the crux of our study.

The Cardiometabolic Index (CMI), defined by the product of the waist-to-height ratio and the triglyceride-to-high-density lipoprotein cholesterol ratio, is an emerging metric of metabolic health (13). Initially introduced by Ichiro Wakabayashi et al. (14), CMI was first validated as a predictor of type 2 diabetes risk. Subsequent investigations have linked CMI with insulin resistance, prediabetes, diabetes, and an elevated risk of cerebrovascular disease (15). Notably, adherence to an anti-inflammatory diet has been shown to reduce both high-sensitivity C-reactive protein (hs-CRP) levels and CMI, suggesting a potential interplay between CMI and systemic immune-inflammatory status (16). Further studies have associated CMI with the severity of atherosclerosis in type 2 diabetes patients, identified it as an independent predictor of cerebral infarction and linked it to all-cause mortality in the elderly, potentially mediated through immune-inflammatory pathways. Despite these associations, the prognostic potential of CMI in predicting long-term outcomes in patients with AIS remains largely unexplored.

In this study, we employed receiver operating characteristic (ROC) curve analysis to compare the predictive accuracy of CMI against lipid metabolism-related markers (TyG and AIP) and an immune-inflammatory marker (LCR) for long-term outcomes in patients with AIS. We specifically assessed the feasibility of CMI as a biomarker for predicting MACEs in this group. Additionally, we explored whether CMI mediates the occurrence of long-term outcomes through systemic immune-inflammatory pathways. This study aims to refine risk stratification and management approaches for patients with AIS.

## Methods

### Study design and patients

This retrospective analysis included 588 patients diagnosed with AIS who underwent emergency thrombolytic therapy at the 904th Hospital of the Joint Logistics Support Force of the People’s Liberation Army of China between October 2016 and October 2019. The exclusion criteria for AIS patients were as follows: (1) administering clopidogrel and aspirin after 4.5 h from symptom onset; (2) Cases were excluded if the following data were missing: admission NIHSS score, neuroimaging confirmation of infarct location and size, or key laboratory parameters including C-reactive protein (CRP), fasting blood glucose, and lipid profile; (3) Attrition within 2 years after leaving the hospital; (4) intracranial hemorrhage and intracranial space-occupying lesions confirmed by CT or MRI; (5) transient ischemic attack (TIA); (6) complications of infection, malignant tumor, severe cardiac insufficiency, liver and kidney diseases, autoimmune diseases; (7) patients with a history of prior stroke or transient ischemic attack (TIA); and (8) death during hospitalization. This study adhered to the principles of the Declaration of Helsinki and was approved by the ethics committees of the Local Ethics Committee of the 904th Hospital of the Joint Logistics Support Force of the People’s Liberation Army of China (Approval No. 20230617). Given the retrospective nature of the study and the use of de-identified patient data, the requirement for written informed consent was waived by the ethics committees.

### Data collection and definitions

Patient information and cerebrovascular disease risk factors were meticulously collected in adherence to specific diagnostic criteria. Hypertension diagnosis followed the 2010 Chinese guidelines, while diabetes determination aligned with the 2013 guidelines for type 2 diabetes control in China. Heart disease included documented conditions like myocardial infarction, angina pectoris, congestive heart failure, or arrhythmia. Upon admission, patients underwent comprehensive assessments, including blood analysis, biochemical evaluations, and various laboratory tests such as hemoglobin, cholesterol levels, inflammatory markers, and blood glucose. The National Institute of Health Stroke Scale (NIHSS) score assessed neurological impairment (17).

At discharge, patients received a regimen comprising medications like Beta-blockers, ACEI/ARBs, calcium channel blockers, aspirin, clopidogrel, statins, and ezetimibe. Composite biomarkers calculated for the study included body mass index (BMI), Cardiometabolic Index (CMI), triglyceride-glucose index (TyG), atherogenic index of plasma (AIP), and lymphocyte-to-C-reactive protein ratio (LCR).

The formula for calculating Cardiometabolic Index (CMI) is shown in the following figure:


$$\mathrm{CMI}=\frac{\text{Triglyceride}\;(\text{mmol}/L)}{\mathrm{HDL}\;\text{cholesterol}\;(\text{mmol}/L)}\times \frac{\text{Waist circumference}\;(\mathrm{cm})}{\text{Height}\;(\mathrm{cm})}$$

Diagnosis of acute ischemic stroke (AIS) relied on persistent or rapidly progressing neurological symptoms with corresponding ischemic changes on imaging. Stroke classifications were confirmed through imaging techniques like magnetic resonance angiography (MRA), CT angiography (CTA), electrocardiography, and echocardiography. AIS cases were categorized based on the TOAST criteria into subtypes like large artery atherosclerosis (LAA), cardio-embolism (CE), small artery occlusion (SAO), stroke of other determined cause (SOD), and stroke of undetermined cause (SUD), covering various origins or incomplete evaluations.

### Long-term cerebrovascular outcomes

Patients received follow-up care via telephone or outpatient services within the initial month post-discharge, followed by subsequent assessments every three months. They or their caregivers were asked about any stroke occurrences or new neurological symptoms. Confirmation of recurrent AIS cases relied on relevant clinical symptoms or neuroimaging data (CT or MRI) assessed by neurologists. Additionally, patients continued their prescribed AIS prevention medications specific to their stroke subtype during the follow-up period. Based on long-term follow-up outcomes, patients were categorized into either the major adverse cerebrovascular event (MACE) group or the non-MACE group. All patients were followed for a maximum of 2 years from the date of enrollment. Patients who were lost to follow-up within this period (i.e., those whose outcome status could not be determined due to loss of contact) were excluded from the analysis. The median follow-up duration was 11 months, reflecting the high event rate in the study cohort, as a considerable proportion of patients experienced the primary endpoint within the first year of follow-up.

The primary endpoint was major adverse cerebrovascular events (MACEs), defined as a composite of (1): recurrent ischemic stroke, confirmed by CT or MRI neuroimaging; (2) hemorrhagic stroke, including intracerebral hemorrhage and subarachnoid hemorrhage confirmed by neuroimaging; and (3) unplanned hospitalization for neurological deterioration, defined as a ≥ 2-point increase in NIHSS score from baseline. All endpoint events were adjudicated based on pre-specified criteria prior to study initiation.

### Statistical analysis

Data were analyzed using SPSS (version 26.0), MedCalc (version 20.022), R (version 4.2.2), and GraphPad Prism (version 9.5.1). The normality of quantitative data was assessed using the Kolmogorov–Smirnov test. Variables that were normally distributed were expressed as mean ± standard deviation and compared using independent *t*-tests, while those not normally distributed were presented as median (interquartile range, 25–75 percentiles) and analyzed using the Mann–Whitney *U* test. Categorical data were expressed as frequencies (percentages) and analyzed using the chi-square test. ROC curves were used to determine the optimal cutoff values for the CMI in predicting the primary endpoint events. The De-Long’s tests compared the predictive capabilities of CMI, TyG, AIP, and LCR. Patients were grouped based on the optimal CMI cutoff to examine the occurrence of primary endpoint events. A four-knot restricted cubic spline (RCS) analysis was employed to explore potential non-linear relationships between the CMI and MACEs, adjusting for a comprehensive set of confounders tailored to specific endpoints. For primary endpoint events, adjustments included age, sex, NIHSS at admission, albumin, hemoglobin, creatinine, WBC, NE, TyG, AIP, and LCR.

The discriminative ability of the model was evaluated using the area under the receiver operating characteristic curve (AUC). AUC values were interpreted according to the criteria described by Hosmer and Lemeshow: 0.5–0.7 indicates poor discrimination, 0.7–0.8 indicates acceptable discrimination, 0.8–0.9 indicates excellent discrimination, and >0.9 indicates outstanding discrimination (18).

Should a non-linear relationship between CMI and cerebrovascular events be identified, further exploration would be conducted using a two-piece Cox proportional hazards regression model, applying a forward stepwise maximum likelihood estimation on either side of the optimal CMI cutoff. Kaplan–Meier survival curves were used to compare event-free survival between groups, with differences assessed using the Log-rank test. All tests were two-tailed, with a significance threshold set at *p* < 0.05.

To avoid over-adjustment bias, LCR was not included as a covariate in the multivariable Cox regression model, given its designated role as a mediator in the subsequent mediation analysis. The multivariable Cox regression and mediation analysis were conducted as independent analytical components with clearly distinct variable assignments.

Additionally, mediation analysis was conducted using the “mediation” package in R, post-adjustment for confounders, to evaluate the mediating role of the composite inflammatory marker LCR in the relationship between CMI and MACEs. A mediation effect was considered present if significant indirect and total effects were observed.

## Results

### Patient enrollment and long-term follow-up

This study enrolled 588 patients diagnosed with AIS from single center cohort after applying specific exclusion criteria that included 588 AIS patients from the 904th Hospital of the Joint Logistics Support Force of the People’s Liberation Army of China, consisting of 485 males and 103 females.

In the single center cohort, the median follow-up duration was 11 months (Interquartile Range, IQR: 3–17 months). During this period, primary endpoint events occurred in 218 cases (37.1%). Individual component incidences were as follows: recurrent ischemic stroke in 115 patients (19.56%), hemorrhagic stroke in 36 patients (6.12%), and hospitalization for neurological deterioration in 67 patients (11.39%).

These findings are summarized in Table 1, which illustrate the distribution and frequency of primary cerebrovascular events across the single center cohort.

**Table 1:** Comparison of baseline characteristics and laboratory parameters associated with long-term major adverse cerebrovascular events (MACEs) between low and high Cardiometabolic Index (CMI) groups.

Basic clinical characteristics	Low CMI level (< 0.80; *n* = 241)	High CMI level (≥ 0.80; *n* = 347)	*P* -value^b^
Demographics			
Age, years	62 (52, 71)	60 (50, 69)	0.038
Male gender, *n* (%)	203 (84.23)	282 (81.26)	0.352
Smoking, *n* (%)	155 (64.32)	218 (62.82)	0.712
Hypertension, *n* (%)	132 (54.77)	216 (62.25)	0.070
Diabetes mellitus, *n* (%)	45 (18.67)	111 (31.99)	<0.001
BMI (kg/m^2^)	23.94 (22.09, 25.45)	24.97 (23.05, 26.67)	<0.001
WC (cm)	97.65 (95.17, 104.02)	102.98 (99.46, 114.33)	<0.001
Height (cm)	170 (165, 174)	170 (163, 173)	0.050
Atrial fibrilation, *n* (%)	55 (22.82)	92 (26.51)	0.309
Admission NIHSS score	4 (2, 6)	4 (2, 7)	0.023
Onset-to-door time (*h*)	3.5 (2.0, 5.0)	3.0 (2.0, 6.0)	0.949
Laboratory parameters			
LPa (ng/dL)	65.35 (23.61, 298.02)	91.52 (30.50, 329.00)	0.122
Lp-PLA2 (ng/dL)	9.16 (1.28, 26.90)	10.44 (1.80, 29.47)	0.558
Hemoglobin (g/L)	139 (128, 148)	140 (127, 153)	0.263
Albumin (g/L)	37.89 ± 4.02	37.756 ± 3.93	0.683^a^
Total cholesterol (mmol/L)	4.26 (3.57, 4.86)	4.46 (3.94, 5.07)	<0.001
Triglyceride (mmol/L)	1.09 (0.89, 1.27)	1.520 (1.05, 2.37)	<0.001
HDL cholesterol (mmol/L)	1.14 (1.00, 1.38)	1.00 (0.84, 1.14)	<0.001
LDL cholesterol (mmol/L)	2.44 (2.02, 2.94)	2.640 (2.22, 3.05)	0.003
C-reactive protein (mg/L)	8.01 (4.17, 11.35)	8.01 (5.04, 19.09)	0.012
Creatinine (μmol/L)	72 (62, 86)	76 (66, 87)	0.010
WBC	9.49 (7.21, 11.70)	10.33 (7.99, 13.14)	0.004
NE	6.57 (4.89, 9.15)	8.17 (6.67, 11.71)	0.013
LY	1.50 (1.07, 2.07)	1.68 (1.20, 2.29)	0.005
PLT	199 (164, 241)	211 (175, 246)	0.027
Uric acid (umol/L)	346 (286, 416)	386 (305, 473)	<0.001
NLR	5.96 (5.15, 7.46)	6.71 (5.36, 9.08)	<0.001
AIP	−0.09 (−0.13, -0.06)	0.25 (0.19, 0.38)	<0.001
TyG	2.81 (2.55, 3.11)	3.50 (3.17, 3.87)	<0.001
PLR	215 (103, 498)	200 (74, 417)	0.207
TOAST			
Localization of AIS, *n* (%)			
Large artery atherosclerosis, *n* (%)	4 (1.66)	11 (3.32)	0.072
Small artery occlusion, *n* (%)	130 (53.94)	172 (49.57)	0.297
Cardioembolism, *n* (%)	40 (16.60)	48 (13.77)	0.182
Other, *n* (%)	33 (13.80)	56 (16.23)	0.147
Undetermined, *n* (%)	34 (14.02)	60 (17.20)	0.556
Post medications			
ACEI/ARBs, *n* (%)	210 (87.14)	316 (91.07)	0.127
CCB, *n* (%)	77 (31.95)	104 (29.97)	0.609
Aspirin, *n* (%)	216 (89.69)	310 (89.34)	0.911
Clopidogrel, *n* (%)	26 (10.79)	31 (8.93)	0.455
Ticagrelor, *n* (%)	208 (86.31)	304 (87.61)	0.644
Statin, *n* (%)	239 (99.17)	343 (98.85)	0.702

CMI, Cardiometabolic Index; TyG, Triglyceride-Glucose Index; AIP, Atherogenic Index of plasma; LCR, lymphocyte-to-C-reactive protein ratio; FBG, fasting blood glucose; BMI, body mass index; ACEI/ARBs, angiotensinogen converting enzyme inhibitor/angiotensinogen receptor blockers; CCB, calcium channel blocker; HDL-C, high-density lipoprotein cholesterol; LDL-C, low-density lipoprotein cholesterol; NE, Neutrophils; LY, lymphocyte; PLT, platelet.

Continuous variables are presented as mean ± SD or median (IQR: 25th–75th percentiles) based on the results of normality testing (Shapiro–Wilk test).

^a^Unpaired Student’s t test. ^b^Mann–Whitney U test and Chi-square test.

### Predictive efficacy of CMI for long-term major adverse cerebrovascular events (MACEs)

**Figure 1:**
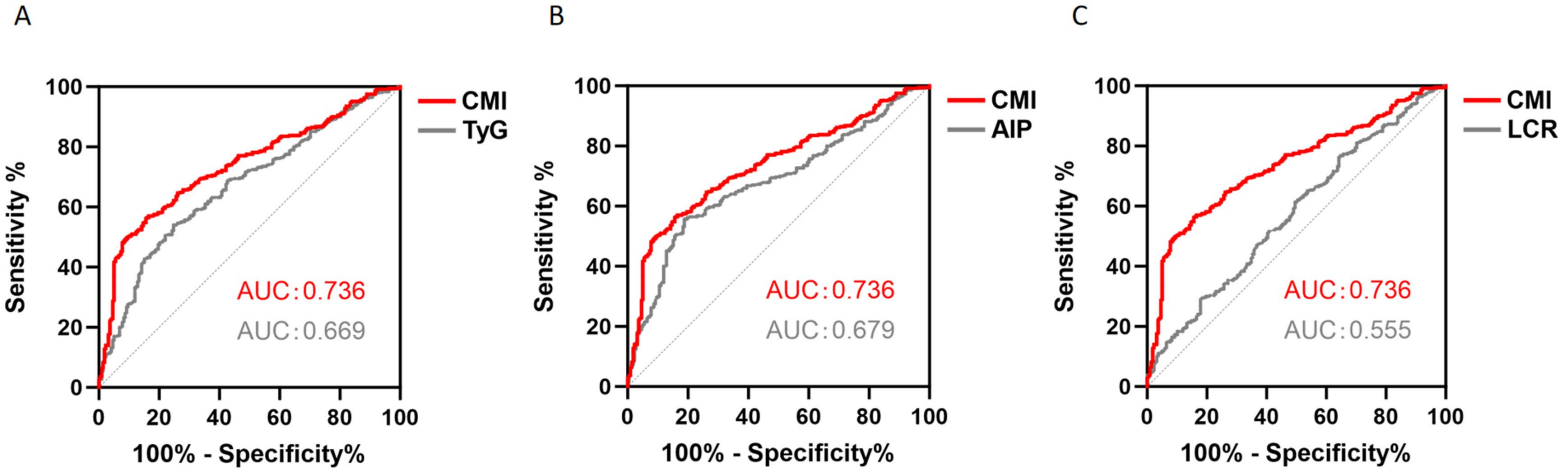
Evaluation of the predictive value of CMI, TyG, AIP, and LCR in predicting long-term outcomes among patients with AIS. **(A)** ROC curve analysis of CMI and TyG. **(B)** ROC curve analysis of CMI and AIP. **(C)** ROC curve analysis of CMI and LCR. CMI, Cardiometabolic Index; TyG, Triglyceride-Glucose Index; AIP, Atherogenic Index of plasma; LCR, lymphocyte to-C reactive protein ratio; NE, neutrophils; WBC, white blood cell; AIS, acute ischemic stroke; ROC, Receiver operating characteristic.

In a detailed analysis of the single center cohort, the predictive values of various biomarkers for long-term outcomes following emergency thrombolytic therapy in patients with AIS were evaluated. The biomarkers assessed included the CMI, TyG, AIP, and LCR. These assessments were conducted using ROC curves, as illustrated in Figure 1. ROC Curve Analysis and Cutoff Values: CMI: The optimal cutoff value was identified as 0.80 (95% CI, 0.699–0.772), demonstrating a sensitivity of 84.4% and a specificity of 56.5%. The area under the curve (AUC) was 0.736, indicating a relatively strong predictive capability. TyG: The best cutoff value was determined to be 3.12 (95% CI, 0.630–0.707), yielding a sensitivity of 75.2% and a specificity of 54.1%, with an AUC of 0.669. AIP: A cutoff value of 0.13 (95% CI, 0.640–0.717) was established, with a sensitivity of 81.2% and a specificity of 55.7%, and an AUC of 0.679. LCR: The cutoff value was set at 101 (95% CI, 0.514–0.596), showing a sensitivity of 35.3% and a specificity of 75.9%, with an AUC of 0.555.

Comparative Analysis Using De-Long’s Test: The De-Long’s test results indicated that the predictive efficacy of CMI for primary endpoint events after intravenous thrombolysis was superior to that of the other biomarkers:

CMI vs. TyG: AUC of 0.736 for CMI is significantly higher than the AUC of 0.669 for TyG (*p* < 0.001). CMI vs. AIP: AUC of 0.736 for CMI is significantly higher than the AUC of 0.679 for AIP (*p* < 0.001). CMI vs. LCR: AUC of 0.736 for CMI is significantly higher than the AUC of 0.555 for LCR (*p* < 0.001).

Based on these findings, CMI emerges as a promising biomarker for predicting long-term adverse cerebrovascular outcomes in patients with AIS.

### Non-linear relationship between CMI and long-term major adverse cerebrovascular events (MACEs)

**Figure 2:**
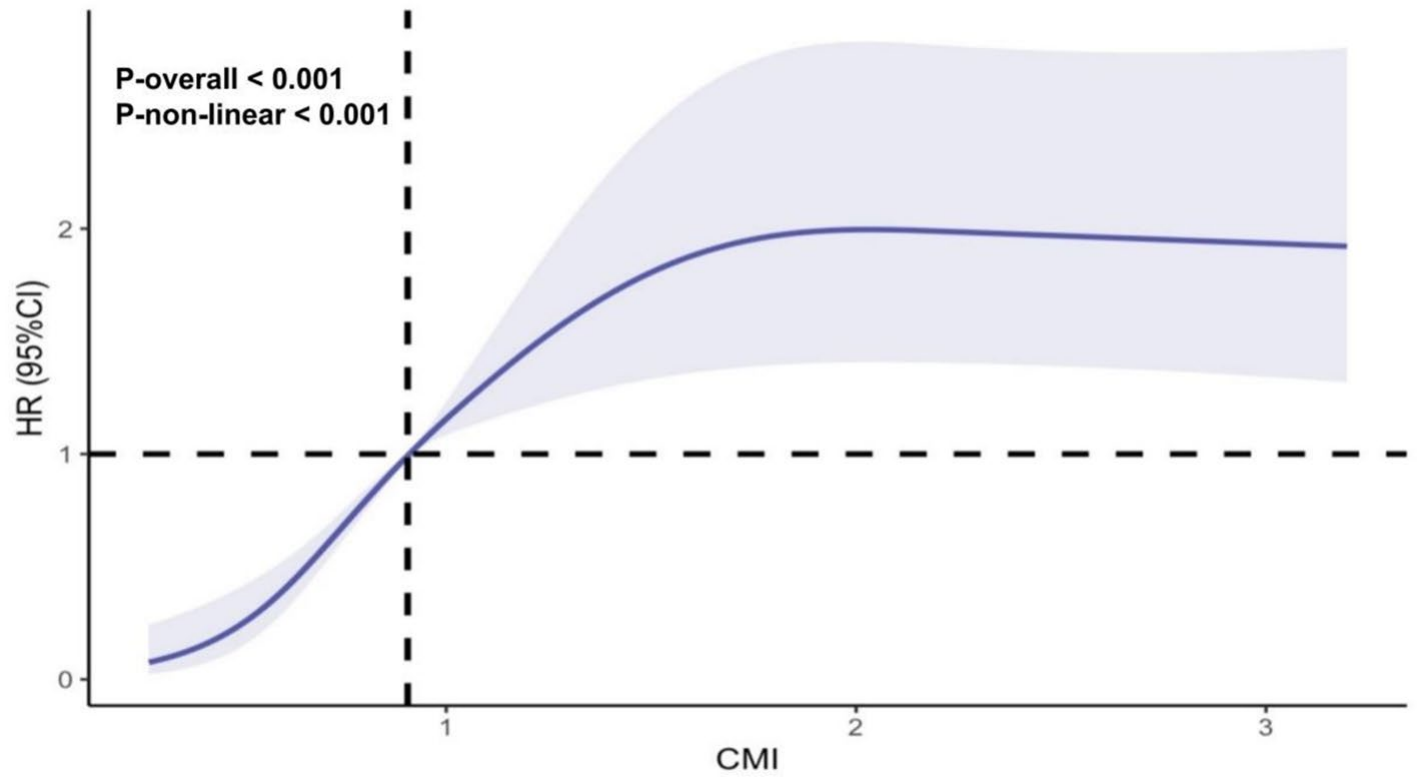
The nonlinear relationship between CMI and the risk of primary endpoint events using a multivariable-adjusted restricted cubic spines model (RCS). A restricted cubic spline (RCS) function was incorporated into the Cox proportional hazards model, adjustments included age, sex, NIHSS at admission, albumin, hemoglobin, creatinine, WBC, NE, TyG, and AIP; Four knots were placed at the 5, 35, 65, and 95% percentiles of the CMI value distribution; The solid blue line delineates the hazard ratio post multivariable adjustment, while the shaded blue area signifies the 95% confidence interval; The X-axis represents the CMI value, and when the CMI value is greater than 0.8, the Y-axis HR value is greater than 1, indicating that as the CMI value increases, the risk of primary endpoint events increases; A likelihood ratio test was used to assess the significance of nonlinearity; HR (95%CI), hazard ratio (95% confidence interval); CMI, Cardiometabolic Index; TyG, Triglyceride-Glucose Index; AIP, Atherogenic Index of plasma; LCR, lymphocyte-to-C-reactive protein ratio; NE, neutrophils; WBC, white blood cell.

In the single center cohort, we evaluated the association between the CMI and the incidence of MACEs following after intravenous thrombolysis in patients with AIS. Utilizing a RCS model, our analysis revealed a significant non-linear relationship between CMI and primary endpoint events, even after adjusting for an extensive array of confounders including age, sex, NIHSS at admission, albumin, hemoglobin, creatinine, WBC, NE, TyG, and AIP (*P*-overall < 0.001, *P*-non-linear < 0.001). These findings, illustrated in Figure 2, underscore the complex and variable influence of metabolic factors, as captured by CMI, on the prognosis of patients with AIS after intravenous thrombolysis. The results highlight the importance of using non-linear models to assess the impact of metabolic indices on cerebrovascular outcomes. Further research is recommended to elucidate the mechanisms underlying these relationships and to validate the predictive utility of CMI in other patient cohorts.

### Basic characteristics of patients

Based on the established optimal cutoff value of 0.80 for the CMI, patients were stratified into two groups: Low CMI (<0.80, *n* = 241) group and High CMI (≥0.80, *n* = 347) group. The analysis revealed that the High CMI group exhibited significantly higher past histories of diabetes, higher BMI, waist circumference (WC), TC, TG, LDL-C, CRP, creatinine, WBC, NE, LY, PLT, uric acid, FBG, TyG, AIP, and NIHSS. Additionally, this group had a higher usage of Beta-blocker compared to the Low CMI group. Conversely, the High CMI group demonstrated lower levels of age and HDL-C, as shown in Table 2.

**Table 2:** Comparison of baseline characteristics and laboratory parameters associated with long-term major adverse cerebrovascular events (MACEs) group and non-MACEs group.

Basic clinical characteristics	Non-MACEs groups, *n* = 370	MACEs groups, *n* = 218	*P* -value^b^
Demographics			
Age, years	59 (50, 68)	64 (53, 72)	0.001
Male gender, *n* (%)	310 (83.78)	175 (80.28)	0.280
Smoking, *n* (%)	240 (64.86)	133 (61)	0.348
Hypertension, *n* (%)	207 (55.95)	141 (64.68)	0.037
Diabetes mellitus, *n* (%)	94 (25.41)	62 (28.44)	0.421
BMI (kg/m^2^)	24.46 (22.55, 25.95)	24.67 (22.84, 26.57)	0.201
WC (cm)	97.65 (95.46, 104.46)	105.96 (100.58, 121.33)	<0.001
Height (cm)	170 (165, 175)	168 (161, 172)	<0.001
Atrial fibrilation, *n* (%)	75 (20.27)	72 (33.03)	<0.001
Admission NIHSS score	4 (2, 7)	5 (3, 6)	<0.001
Onset-to-door time (*h*)	3 (2, 6)	3 (2, 6)	0.604
Follow-up duration, months	11 (6, 18)	11 (7, 16)	0.233
Laboratory parameters			
LP (a) (ng/dL)	65.28 (23.18, 298.68)	111.95 (36.30, 336.50)	0.007
Lp-PLA2 (ng/dL)	8.80 (1.356, 26.22)	11.05 (1.79, 33.79)	0.164
Hemoglobin (g/L)	140 (130, 152)	136 (122, 151)	0.007
Albumin (g/L)	38.07 ± 3.86	37.38 ± 4.10	0.043^a^
Total cholesterol (mmol/L)	4.39 (3.70, 4.94)	4.42 (3.89, 5.02)	0.247
Triglyceride (mmol/L)	1.34 (1.06, 1.89)	1.80 (1.39, 2.12)	<0.001
HDL cholesterol (mmol/L)	1.05 (0.91, 1.25)	1.06 (0.90, 1.17)	0.050
LDL cholesterol (mmol/L)	2.57 (2.10, 3.00)	2.56 (2.19, 2.97)	0.788
C-reactive protein (mg/L)	8.01 (5.09, 11.71)	8.01 (4.09, 21.51)	0.059
Creatinine (μmol/L)	73 (63, 84)	80 (67, 91)	<0.001
WBC	9.74 (7.62, 12.39)	10.37 (7.88, 12.87)	0.161
NE	6.95 (5.09, 9.22)	7.28 (5.20, 10.26)	0.060
LY	1.62 (1.17, 2.23)	1.53 (1.09, 2.03)	0.075
PLT	208 (170, 247)	206 (166, 235)	0.227
Uric acid (umol/L)	367 (290, 445)	378 (305, 452)	0.152
NLR	6.16 (5.18, 7.77)	6.70 (5.44, 9.11)	0.008
AIP	0.10 (−0.30, 0.25)	0.21 (0.15, 0.33)	<0.001
TyG	3.08 (2.70, 3.53)	3.43 (3.11, 3.85)	<0.001
PLR	216 (102, 437)	183 (66, 424)	0.025
TOAST			0.060
Localization of AIS, *n* (%)			
Large artery atherosclerosis, *n* (%)	6 (1.62)	13 (5.96)	0.004
Small artery occlusion, *n* (%)	189 (51.08)	113 (51.83)	0.860
Cardioembolism, *n* (%)	58 (15.68)	26 (11.93)	0.210
Other, *n* (%)	100 (27.03)	59 (27.06)	0.733
Undetermined, *n* (%)	17 (4.59)	7 (3.21)	0.363
Post medications			
ACEI/ARBs, *n* (%)	41 (11.08)	33 (15.14)	0.152
CCB, *n* (%)	157 (42.43)	99 (45.41)	0.481
Aspirin, *n* (%)	366 (98.92)	214 (98.17)	0.896
Clopidogrel, *n* (%)	358 (96.76)	209 (95.87)	0.938
Ticagrelor, *n* (%)	12 (3.24)	9 (4.13)	0.768
Statin, *n* (%)	367 (99.19)	215 (98.62)	0.349

CMI, Cardiometabolic Index; TyG, Triglyceride-Glucose Index; AIP, Atherogenic Index of plasma; LCR, lymphocyte-to-C-reactive protein ratio; FBG, Fasting blood glucose; BMI, body mass index; ACEI/ARBs, angiotensinogen converting enzyme inhibitor/angiotensinogen receptor blockers; CCB, calcium channel blocker; HDL-C, high-density lipoprotein cholesterol; LDL-C, low-density lipoprotein cholesterol; NE, neutrophils; LY, lymphocyte; PLT, platelet.

Continuous variables are presented as mean ± SD or median (IQR: 25th–75th percentiles) based on the results of normality testing (Shapiro–Wilk test).

^a^Unpaired Student’s t test. ^b^Mann–Whitney U test and Chi-square test.

### Relationship with major adverse cerebrovascular events (MACEs)

In the single center cohort, patients were stratified based on the occurrence of MACEs into two groups: those with MACEs (*n* = 218) and those without (Non-MACEs, *n* = 370). The MACEs group exhibited higher age, a greater prevalence of hypertension, elevated levels of TG, creatinine, TyG, AIP. They also had higher NIHSS, but lower levels of hemoglobin, albumin, and LCR compared to the Non-MACEs group, as shown in Table 2.

**Figure 3:**
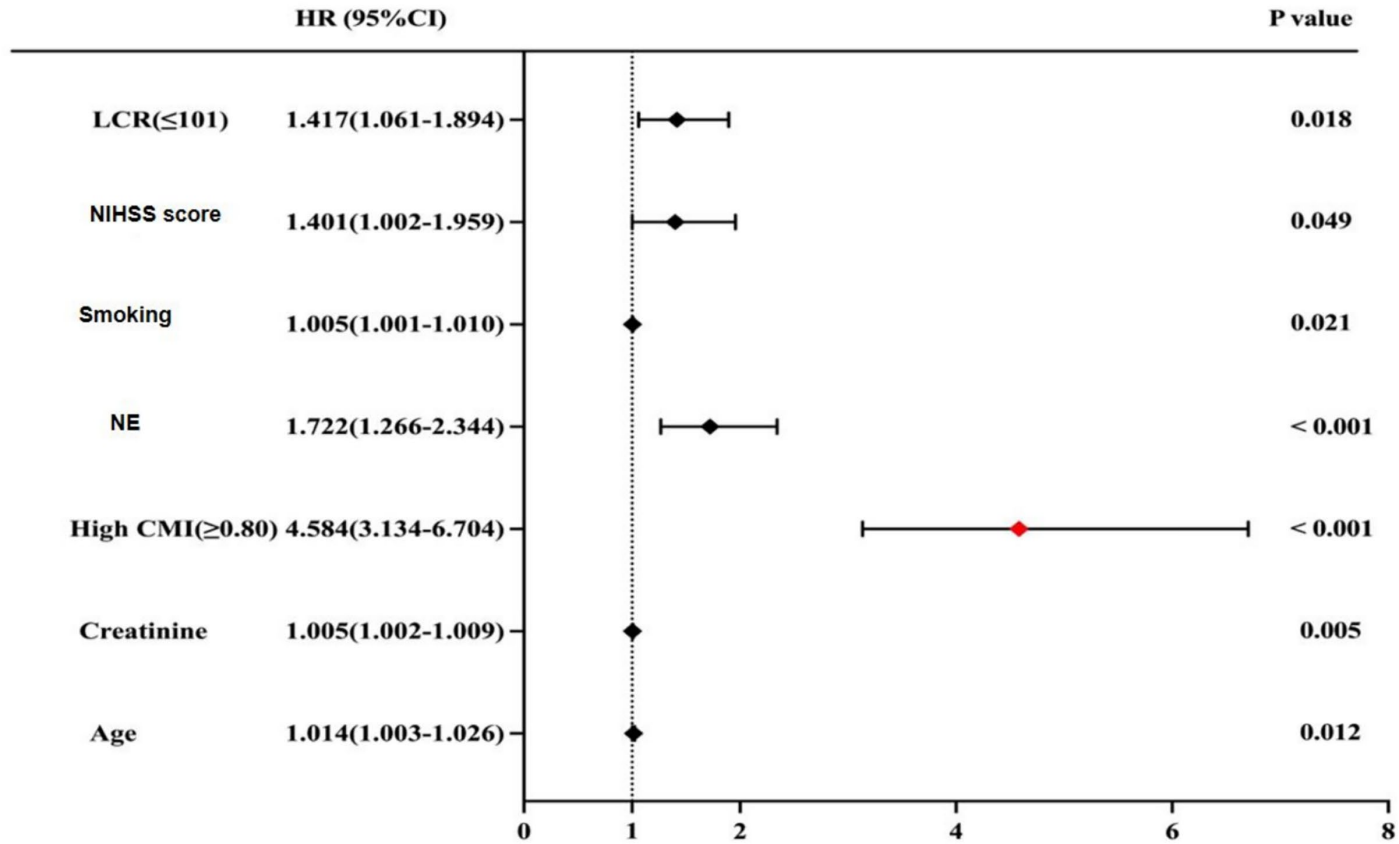
Multivariate Cox regression analysis models for independent predictors of primary endpoint events. Multivariate Cox regression analysis was used to screen for adverse risk factors for long-term primary endpoint events in AIS patients, adjustments included age, Hypertension, albumin, hemoglobin, creatinine, triglyceride, NE, NIHSS score, TyG, AIP, and LCR. Confirmed LCR (≤ 101), NIHSS score, smoking, NE, High CMI (≥ 0.80), creatinine, and age were identified as independent risk factors for the primary endpoint events; HR (95%), hazard ratio (95% confidence interval); CMI, Cardiometabolic Index; TyG, triglyceride-glucose index; AIP, Atherogenic index of plasma; LCR, lymphocyte-to-C-reactive protein ratio; NE, Neutrophils.

Multivariable Cox regression analysis identified several independent risk factors for primary endpoint events following emergency thrombolytic therapy in patients with AIS: low LCR (≤101), higher NIHSS, increased creatinine levels, older age, and high CMI (≥0.80). Notably, a high CMI was associated with a hazard ratio (HR) of 4.584 (95% CI: 3.134–6.704, *p* < 0.001), as depicted in Figure 3 and Table 3.

**Table 3:** Univariable analyses and multivariable models for independent predictors of long-term outcomes.

Basic clinical characteristics	Univariate analysis		Multivariable analysis	
	HR (95% CI)	*p* value	HR(95% CI)	*P* value
LCR (≤101)	2.451 (1.107–5.427)	0.027	1.417 (1.061–1.894)	0.018
NIHSS score	2.648 (1.296–5.411)	0.008	1.410 (1.002–1.959)	0.049
Smoking	1.986 (1.034–3.692)	<0.001	1.005 (1.001–1.010)	0.021
NE	2.437 (1.998–3.007)	<0.001	1.722 (1.266–2.344)	<0.001
High CMI	5.523 (3.798–6.997)	<0.001	4.584 (3.134–6.704)	<0.001
Creatine	1.789 (1.001–2.934)	0.034	1.005 (1.002–1.009)	0.005
Age	2.147 (1.136–2.986)	0.003	1.014 (1.003–1.026)	0.001

CMI, Cardiometabolic Index; LCR, Lymphocyte-to-C-reactive protein ratio; NE, neutrophils.

**Figure 4:**
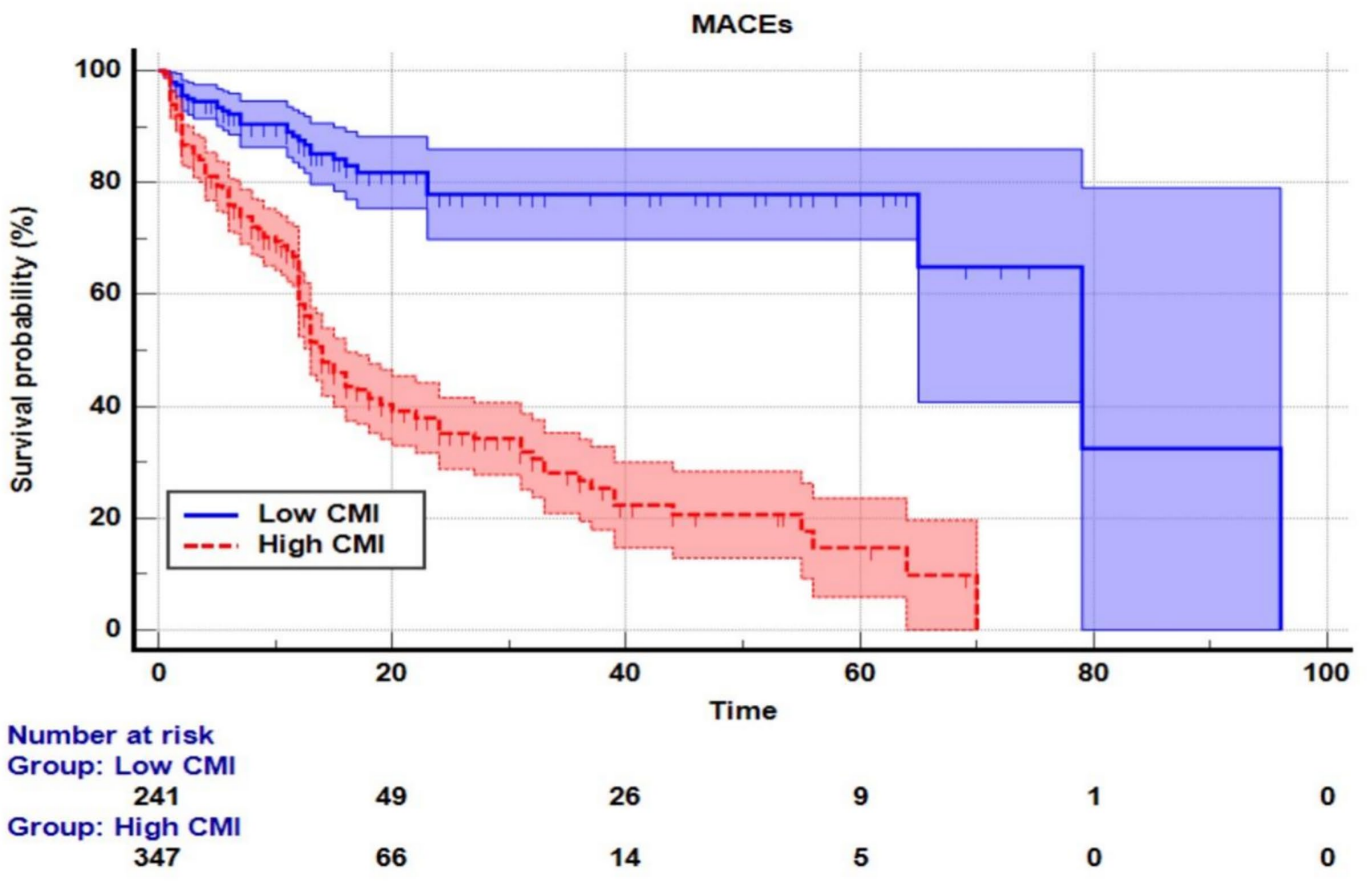
Kaplan–Meier survival analysis. Patients with a high CMI group (CMI ≥ 0.80) had much lower event-free survival at long-term follow-up than patients with a Low CMI group (CMI < 0.80). 588 patients were divided into two groups based on a CMI value of 0.8, with 347 patients in the High CMI group (≥ 0.80) and 241 patients in the Low CMI group (CMI < 0.80). Kaplan–Meier survival curves were plotted based on the long-term follow-up endpoint events of the two groups. The results showed that the long-term event free survival rate of patients in the Low CMI group (CMI < 0.80) was higher than that in the High CMI group (≥ 0.80). MACEs, primary endpoint events; CMI, Cardiometabolic Index.

The Kaplan–Meier survival curves indicate that patients in the high CMI (≥0.80) group exhibited significantly lower long-term event-free survival rates (log-rank *p* < 0.001) (Figure 4).

### Mediation effect of lymphocyte-to-C-reactive protein ratio (LCR)

**Figure 5:**
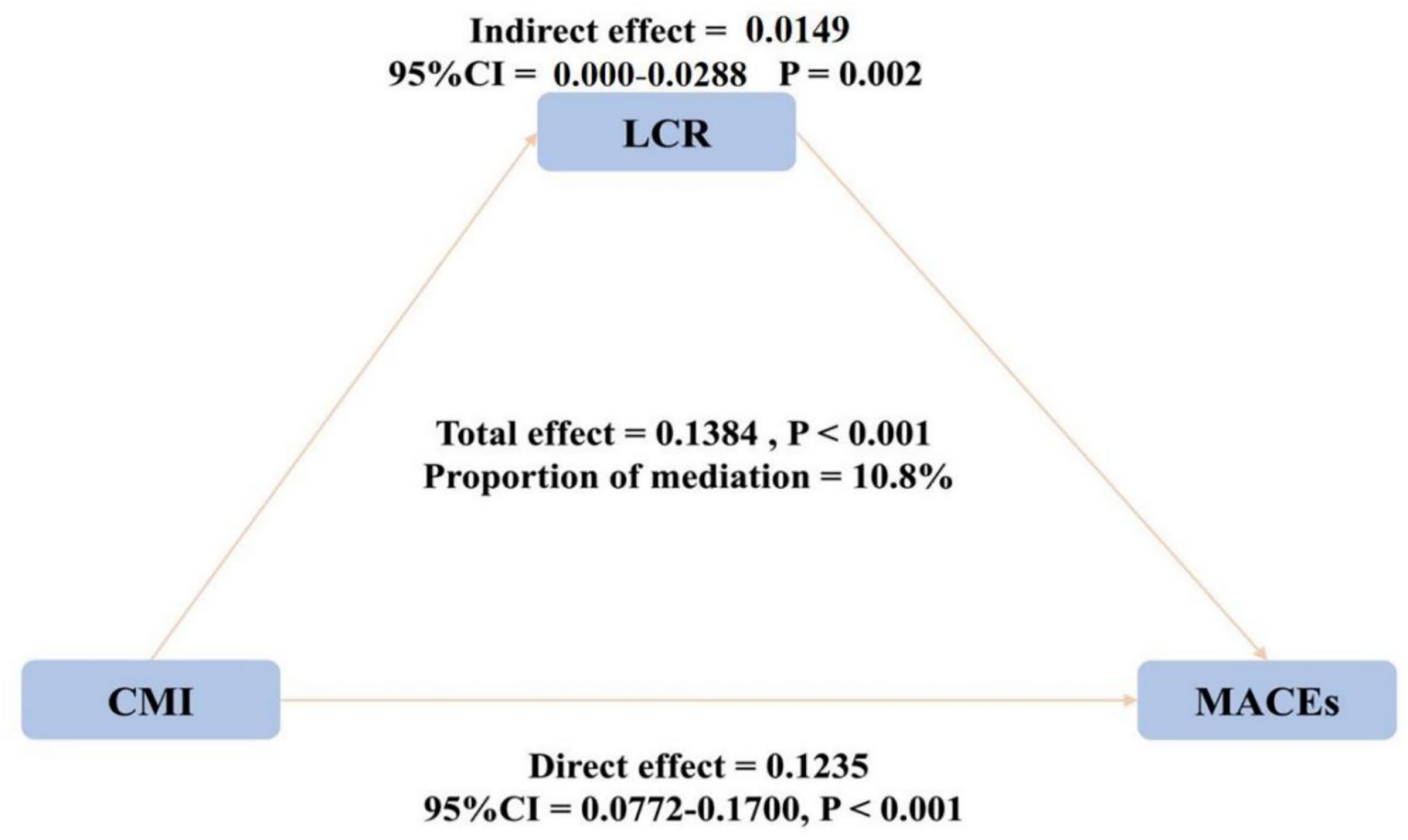
Analysis of how inflammation mediates the associations between CMI and long-term outcomes. Mediation analysis revealed that the LCR mediated 1.49% of the association between the CMI and primary endpoint events, indicating that CMI impacts outcomes not only directly but also indirectly through its influence on LCR. MACEs, primary endpoint events; CMI, Cardiometabolic Index; LCR, lymphocyte-to-C-reactive protein ratio.

Mediation analysis revealed that the LCR mediated 1.49% of the association between the CMI and primary endpoint events, indicating that CMI impacts outcomes not only directly but also indirectly through its influence on LCR, as shown in Figure 5.

## Discussion

In this study, we identified and evaluated the feasibility of CMI as a novel prognostic biomarker for long-term adverse events following after intravenous thrombolysis in patients with AIS.

Thus, CMI emerges as a potential biomarker for poor prognosis following emergency thrombolytic therapy in patients with AIS. Additionally, the mediation analysis indicated that CMI influenced the occurrence of primary endpoint events through systemic inflammatory status, as reflected by the LCR. This underscores the multifaceted role of CMI in modulating both metabolic and inflammatory pathways, which are critical in the prognosis of patients with AIS after intravenous thrombolysis.

Initially, we assessed the predictive efficacy of the CMI compared to traditional lipid metabolism and inflammatory composite indices (TyG, AIP, and LCR) using ROC curves in the single center cohort. The results demonstrated that CMI showed superior discriminative ability compared to these traditional indices in predicting long-term outcomes in our cohort. Further analyses, employing RCS and Cox regression models adjusted for confounders, revealed a nonlinear relationship between CMI and primary endpoint events. Notably, a high CMI (≥0.80) emerged as an independent risk factor for adverse cerebrovascular outcomes. Additionally, multivariable Cox regression analysis further identified advanced age and NIHSS at admission as independent risk factors for primary endpoint events, aligning with findings from previous studies.

Recent research has enriched our understanding of the CMI and its association with cerebrovascular diseases, linking it to conditions such as insulin resistance, newly diagnosed diabetes, atherosclerosis in patients with type 2 diabetes, hypertension-related cerebrovascular diseases, stroke, biological aging, and increased all-cause mortality in the elderly (19–21). Despite these associations, studies focusing on CMI as a predictor of long-term cerebrovascular adverse events following emergency thrombolytic therapy in patients with AIS remain limited.

The potential mechanisms by which CMI serves as a biomarker for MACEs in patients with AIS are multifaceted. Firstly, as a cardiac metabolic index related to insulin resistance, CMI is directly involved in endothelial dysfunction, oxidative stress, and systemic inflammatory responses, which accelerate the progression of cerebral artery atherosclerosis and impact long-term outcomes (9). Secondly, CMI, composed of lipid metabolism parameters (TG, HDL-C) and obesity-related metrics (WC, height), reflects the management of LDL-C and other lipid-related indices after intravenous thrombolysis, which can significantly improve patient outcomes. Additionally, the product of WC and height, serving as a measure of central obesity [Waist-to-Height Ratio (WHtR)], independently predicts risks of myocardial infarct and ischemic stroke, suggesting that CMI may influence cerebrovascular events through its composite reflection of lipid metabolism and WHtR. Finally, adherence to an anti-inflammatory diet has been shown to reduce systemic inflammatory states and CMI levels, indicating a possible linkage between CMI and systemic inflammation. Research by Xu et al. (20) has demonstrated that systemic inflammation partially mediates the relationship between CMI and insulin resistance as well as diabetes onset, and also plays a role in the association between CMI and all-cause mortality in the elderly. Similarly, Previews study found that the LCR, a composite marker of systemic inflammation, partly mediated the relationship between CMI and long-term adverse events in patients with AIS, suggesting that CMI may influence adverse cerebrovascular outcomes through its impact on systemic immune-inflammatory states.

Compared to traditional cerebrovascular risk markers—TyG for insulin resistance, AIP for lipid metabolism, and LCR for inflammatory status—CMI integrates these factors, potentially offering superior predictive capability for long-term MACEs in patients with AIS undergoing after intravenous thrombolysis. Thus, CMI could better facilitate the prognostication of these patients, aiding therapeutic decision-making and improving clinical outcomes and follow-up.

This study has several limitations that warrant consideration. Firstly, as a retrospective analysis conducted across single center medical institutions, the relatively small sample size necessitates further research involving multiple centers to increase the sample size. Such expansion is crucial to validate the stability and applicability of the CMI as a novel predictor for long-term outcomes in patients with AIS. Secondly, although mediation analysis suggested that systemic inflammatory status might mediate the relationship between CMI and endpoint events, the ongoing debate regarding the efficacy of anti-inflammatory treatments in improving long-term outcomes in patients with AIS remains unresolved. Future prospective studies should explore whether dietary modifications or anti-inflammatory medications can enhance clinical outcomes for these patients. Lastly, while this study focused on lipid and metabolic markers, the development of more precise biomarkers through advanced methodologies such as machine learning remains a priority. Additionally, NIHSS scores were only collected at admission and were not systematically recorded at follow-up, which limits our ability to assess longitudinal neurological recovery in this retrospective cohort. Our team is committed to refining these predictive models to improve their sensitivity, specificity, and overall diagnostic efficacy.

## Conclusion

In summary, the CMI may serve as an independent predictor of MACEs in patients with AIS following after intravenous thrombolysis. CMI represents a feasible and promising biomarker for predicting adverse outcomes in AIS patients following after intravenous thrombolysis.

## Data Availability

The original contributions presented in the study are included in the article/supplementary material, further inquiries can be directed to the corresponding author/s.
